# Reflections of well-being: navigating body image, chronic energy deficiency, and nutritional intake among urban and rural adolescents

**DOI:** 10.3389/fnut.2024.1346929

**Published:** 2024-05-01

**Authors:** Cica Yulia, Delita Septia Rosdiana, M. Muktiarni, Desyane Ramadhina Sari

**Affiliations:** ^1^Culinary Art Education Study Programme, Universitas Pendidikan Indonesia, Bandung, Indonesia; ^2^Nutrition Department, Universitas Pendidikan Indonesia, Bandung, Indonesia

**Keywords:** adolescents, body image, chronic energy deficiency (CED), nutritional intake, rural, urban

## Abstract

**Background:**

Adolescent growth and development is a period of very specific nutritional problems. As a result of poor growth and development, 36.3% of adolescents in Indonesia are at risk of developing CED. The purpose of this study was to determine the description of body image, the incidence of Chronic Energy Deficiency (CED), and nutritional intake in adolescents in urban and rural areas.

**Methods:**

This study used a descriptive quantitative design with a cross-sectional study conducted in Bandung and Sumedang on 387 adolescents aged 13–15 years. The instruments used in this study were body image questionnaire Figure Rating Scale (FRS) method, 2 × 24-h food recall, and anthropometry for Measuring mid upper arm circumference (MUAC).

**Results:**

Results of this study showed that more than half of adolescents in urban (54.0%) and rural areas (61.7%) were at risk of CED, had negative body image perceptions in urban (69.1%) and rural areas (62.3%), and underconsumption of macronutrients in both urban and rural adolescents.

**Conclusion:**

Most adolescents in urban and rural areas still consume less energy, carbohydrates, and protein. Perceived body image and nutrient intake contribute to the incidence of CED in adolescents.

## 1 Introduction

The growth period in adolescents is a time of very specific nutritional problems. The growth and development of puberty, linear growth, and the occurrence of neurodevelopmental changes are strongly influenced by nutritional intake that meets the needs of adolescents (1). Adequate intake of nutrients such as carbohydrates, protein, iron, calcium and other micronutrients will increase in adolescence and is strongly influenced by diet. Not fulfilling nutrients will hinder optimal growth and development and cause nutritional problems in adolescents (2). Nutritional problems often occur due to unbalanced nutrient intake and can cause chronic energy deficiency (CED) and anemia due to iron deficiency (3).

Chronic energy deficiency (CED) is a condition in which an adolescent girl or woman suffers from nutritional deficiencies (energy and protein) that occur over a long period of time or even years (4). A person is diagnosed as being at risk of developing CED if the mid upper arm circumference (MUAC) is <23.5 cm. Adolescents who experience CED are often caused by low macronutrient intake, especially energy and protein, which can also contribute to low micronutrient intake. In the case of adolescent girls who suffer from CED, if not treated properly, it will be sustained and lead to low academic performance. Malnutrition is a major contributing factor to low academic performance and school enrollment (5). In addition, CED also affects pregnancy which can trigger other diseases such as anemia, premature labor, birth of low-weight babies (LBW), giving birth to stunted children, and even risking death (6).

The prevalence of chronic energy deficiency (CED) among adolescent girls (aged 15–19 years) in Indonesia in 2020 has increased from 2018, which was 33.5 to 36.3% (7). Based on this prevalence, an estimated 7.5 million or 3–4 out of 10 adolescent girls in Indonesia suffer from anemia. Another impact of CED on adolescents is the inhibition of growth and development, cognitive abilities, and susceptibility to infectious diseases (8). There are several factors that influence the occurrence of CED in adolescents, direct and indirect factors. Direct factors are infectious diseases and age while indirect factors are knowledge of nutrition and physical activity of adolescents (9). In developing countries, the main influencing factor is infectious diseases caused by poor diet and nutritional intake.

In adolescence, there is a growing awareness of appearance (body image) depending on the opinions of others and the environment. Dissatisfaction with their appearance occurs if their body shape looks fatter or feels too thin than usual, causing adolescents to be more likely to restrict their diet (10, 11). Body image in adolescents greatly affects their diet, including the selection of food ingredients, meal frequency, and nutritional intake (12). This study aims to see how the description of body image, CED, and nutritional intake in adolescents in urban and rural areas.

Health problems in the adolescent phase need more attention due to the many factors that can affect their well-being. The increasing prevalence of adolescent CED in Indonesia indicates the need for more serious handling to prevent long-term health problems. At this age, adolescents begin to choose the food they consume and pay attention to their body shape according to their self-perception and social environment (13). Adolescents who have a negative body perception are likely to make the wrong diet and lead to nutritional deficiencies (14). The aims of this study is to:

1. Analyze how body image perceptions among adolescents in urban and rural areas
2. Analyze the nutritional status and prevalence of CED among adolescents in urban and rural areas
3. Analyze the macronutrient intake of adolescents in urban and rural areas

## 2 Materials and methods

### 2.1 Study population

**Figure 1:**
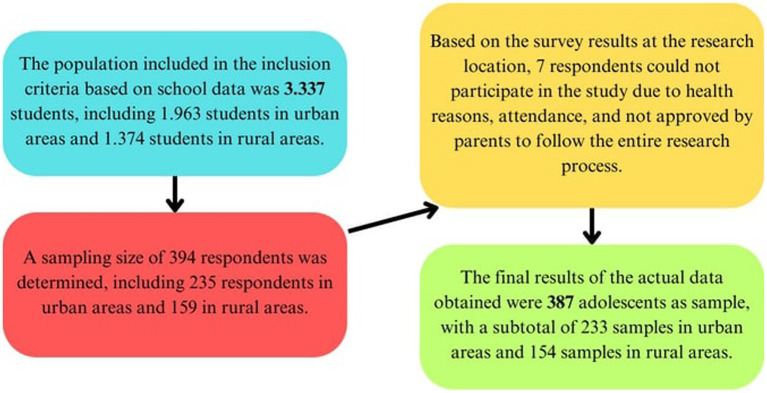
Research population and sample.

The population in this study were junior high school students in 5 schools in urban and 5 schools in rural areas. Research location were determined based on criteria and school clusters in each urban and rural areas. To represent the heterogenization of the respondents, the schools were determined based on economic status, school location, and type of school, either public or private school. Economic status was considered as one of the criteria because it can affect the diet of adults and also affect the diet of their children. Based on the research, this may indicate that wealthier people can consume a healthier and more diverse food which affects nutritional status. The sampling process used random sampling techniques, with the inclusion criteria of adolescents aged 13–15 years and willing to participate in the entire research program by filling out informed consent. This sampling was determined using the Slovin formula with an error rate of 5%. Based on the sampling results and the actual situation in the location, 387 students were obtained as respondents in this study (Figure 1).

### 2.2 Policies and publication ethics

The research was conducted in August–September 2023, and has a publication ethics code with Number LB.01.03/6/317/2023 from Ministry of Health of the Republic of Indonesia Directorate General of Health Workers of Mataram Health Polytechnic.

### 2.3 Measurement and definition

The design of this study was descriptive quantitative research with a cross-sectional approach with a research focus to determine the description of body image, CED, and macronutrient intake in adolescents in urban and rural areas. The research instruments used in this study were body image questionnaire Figure Rating Scale (FRS) method, 2 × 24-h food recall, and anthropometry to determine age, nutritional status and MUAC. The data results were tested statistically to describe the frequency of each variable. Here are the research steps in this study:

#### 2.3.1 Preparation of the research instruments

This research uses various types of instruments that can cover the required data results.

##### 2.3.1.1 Figure rating scale

Figure Rating Scale (FRS) is a psychological assessment instrument used to measure perceptions of body beauty and body image, and can be used in research related to body image, self-esteem, and disorder. This instrument consists of illustrative images of various forms of male and female body sizes equipped with 11 questions related to ideal body perceptions. In this study, the instrument was used to assess how adolescents aged 13–15 years perceived their body image.

##### 2.3.1.2 Food recall 2 × 24 h

Food recall is an assessment instrument used to collect information through interviews about food consumed by individuals during a certain period. In this study, the food recall instrument contains meal times, food and drinks consumed, including the amount and type of food. This instrument is used to see how nutritional intake, especially macronutrients, in adolescents aged 13–15 years. This study used a 2 × 24 h food recall instrument on weekdays and weekends, in order to represent the overall nutritional intake and the resulting data is more qualified.

##### 2.3.1.3 Anthropometry

This instrument was prepared to determine the nutritional status of respondents including gender, age, weight, height, and mid upper arm circumference (MUAC). MUAC is one of the anthropometric indicators used to assess the nutritional status of chronic energy deficiency (CED) in adolescents. The equipment used to measure anthropometry is a SAGA stadiometer to measure height, Omron HBF-375 Bioelectrical Impedance Analysis (BIA) to measure body composition, especially body weight, and an automatic meter to measure upper arm circumference.

#### 2.3.2 Instrument trials

The results of the instrument preparation carried out by the research team were then tested on adolescents aged 13–15 years. The instrument testing process was conducted with the aim of knowing how long it takes to do the interview process and anthropometry to respondents, as well as to find out whether the instrument compiled has represented the data needed by the research team. The instrument trial was conducted in one school with 8 adolescents who became trial samples. The results of the instrument trial was the need to make improvements to the interview procedure and anthropometric measurement process, because during the trial it took quite a long time and was less efficient. As the instrument was compiled based on various previous studies, the instrument was in accordance with the aims of this study.

#### 2.3.3 Cooperation with the schools

Based on the research location that had been determined, the research team met with the school and conveyed the purpose of the research. The research team conveyed the entire research process and methods used, discussed the place and time of data collection, and the necessary administration. The permit documents were fully completed by the research team so that the cooperation process went well.

#### 2.3.4 Providing informed consent

This research requires informed consent approved by the parents of adolescents who become research samples. The informed consent was provided and delivered to the students through the school office. Students who become samples are required to bring the informed consent during the data collection process so that it is confirmed that they can take part in the entire research process.

#### 2.3.5 Data collection process

Data collection was carried out by enumerators who had a background in nutrition education and had participated in a training or trial process before data collection, so that they were able to comprehend and master the used instruments. The instrument used was in paper form and also digital using google form. All students who became samples were asked to fill out the Figure Rating Scale (FRS) instrument via their smartphones, followed by the anthropometric process and food recall interviews directly with each sample. The food recall interview process is carried out twice according to the data needed.

#### 2.3.6 Data cleaning process

The data that has been collected was inputted using Microsoft excel and a cleaning process was conducted to find out if there were any errors or miss-entries during the data collection process. This also aims to facilitate the analysis process.

#### 2.3.7 Data analysis process

The data analysis process was carried out by professional assistance analyzed using IBM SPSS Statistics 27 software. Data analysis includes the frequency of age, gender, nutritional status, body image perception classification, and the average value of macronutrient consumption (median) of adolescents aged 13–15 years.

## 3 Results

### 3.1 Characteristics and nutritional status

Adolescence is a transitional period from childhood to adulthood, starting from the age of 10–13 years and ending at the age of 18–22 years (15). Table 1 shows that in urban areas, the percentage of research respondents were male (48.5%) and female (51.5%), while in rural areas the research respondents were male (46.8%) and female (53.2%). Urban areas respondents aged 13 years (36.9%), 14 years (42.1%), 15 years (21.0%) and in rural areas respondents aged 13 years (48.7%), 14 years (41.6%), 15 years (9.7%). Comparison based on the results of data analysis, more than half of the female respondents were 14 years old in urban areas and 13 years old in rural areas. The classification of economic status was determined by referring to the average income in urban and rural areas. Based on economic status, adolescent parents in urban areas had an income of ≤3,500,000 (50.6%) and in rural areas (62.3%). Table 1 shows that the percentage of adolescents at risk of developing CED is higher in rural areas (61.7%) and urban areas (54.0%).

**Table 1:** Sociodemographic characteristics and nutritional status (*n = 387*).

Variable	Urban (*n* = 233)	Rural (*n* = 154)
	n (%)	n (%)
Gender		
Male	113 (48.5)	72 (46.8)
Female	120 (51.5)	82 (53.2)
Age		
13 years	86 (36.9)	75 (48.7)
14 years	98 (42.1)	64 (41.6)
15 years	49 (21.0)	15 (9.7)
Economic status		
> IDR3,500,000	115 (49.4)	58 (37.7)
≤ IDR3,500,000	118 (50.6)	96 (62.3)
Nutritional status		
Normal	107 (45.9)	59 (38.3)
CED (<23,5 cm)	126 (54.0)	95 (61.7)

### 3.2 Body image perception

Body image perception was classified into two categories, namely positive and negative perceptions. Positive body image is associated with bodily satisfaction and acceptance, whereas negative body image is associated with unhappiness and the desire for one’s body to change (16). Based on Table 2, adolescents in urban areas had a negative body image (69.1%) and a positive body image (30.9%). In rural areas, adolescents who have a negative body image (62.3%) and positive (37.7%). The results of this study are in line with the research of Fitria et al. (17), stating that as many as 73.63% of adolescents in Yogyakarta have a negative body image. In contrast to research in Italy, men and women showed a good perception of their bodies, and showed little tendency to overestimate one’s weight status (*p* = 0.073) (18).

**Table 2:** Body image perception (*n = 387*).

Body image perception	Urban (*n* = 233)	Rural (*n* = 154)
	n (%)	n (%)
Positive	72 (30.9)	58 (37.7)
Negative	161 (69.1)	96 (62.3)

### 3.3 Nutritional intake

The nutrient intakes analyzed in this study were macronutrients including energy, protein, fat and carbohydrate intake. Nutritional Adequacy Rate (NAR) was classified based on the provisions set by the Indonesian minister of health, in the regulation of the minister of health of the Republic of Indonesia No. 28 of 2019. Adolescent boys aged 13–15 must consume sufficient energy (2,400 kcal), protein (70 g), fat (80 g) and carbohydrates (350 g). Adolescent girls aged 13–15 should consume sufficient energy (2050 kcal), protein (65 g), fat (70 g) and carbohydrates (300 g). Adolescent are considered fulfilled if they reach the NAR. Based on Table 3, the energy intake in urban areas was found to be less than the requirement based on the NAR, with a percentage of less (89.3%) and adequate (10.7%). As well as, in rural areas, adolescents’ energy intake was categorized as less (80.5%) and adequate (19.5%). Protein intake of adolescents in urban areas was categorized as less (87.6%), adequate (12.4%), and in rural with percentages of less (82.5%) and sufficient (17.5%). Fat intake was categorized as deficient with percentages of adolescents in urban (69.0%) and rural areas (79.9%). The consumption of carbohydrate intake of adolescents was also categorized as insufficient with percentages in urban (91.8%) and rural areas (85.7%).

**Table 3:** Macronutrients intake (*N = 387*).

Macronutrients Intake	Urban (*n* = 233)	Rural (*n* = 154)
	n (%)	n (%)
Energy		
Less	208 (89.3)	124 (80.5)
Adequate	25 (10.7)	30 (19.5)
Protein		
Less	204 (87.6)	127 (82.5)
Adequate	29 (12.4)	27 (17.5)
Fat		
Less	161 (69.0)	123 (79.9)
Adequate	72 (31.0)	31 (20.1)
Carbohydrate		
Less	214 (91.8)	132 (85.7)
Adequate	19 (8.2)	22 (14.3)

### 3.4 Correlation between nutritional status and influencing variables

According to Table 4, result showed there was no significant correlation between the variables. It was found that adolescents in urban areas who had negative body image perceptions had the highest percentage of CED (50.5%). In rural areas, adolescents who had a negative body image and were diagnosed with CED had a higher percentage compared to adolescents in urban areas (60.6%). Despite the lack of correlation between the variables, the data in Table 4 showed that in both urban and rural areas, adolescents who had negative body image perceptions and also consumed less macronutrients were significantly more affected by CED.

**Table 4:** Correlation between variables (*N = 387*).

	Nutritional status					
	Urban (*n* = 233)			Rural (*n* = 154)		
	CED	Normal	Sig.	CED	Normal	Sig.
	n (%)	n (%)		n (%)	n (%)	
Body image perception						
Positive	25 (75.7)	8 (24.3)	0.007	18 (66.6)	9 (33.4)	0.558
Negative	101 (50.5)	99 (49.5)		77 (60.6)	50 (39.4)	
Macronutrients intake						
Energy						
Less	113 (54.3)	95 (45.7)	0.993	77 (62)	47 (38)	0.386
Adequate	13 (52)	12 (48)		18 (60)	12 (40)	
Protein						
Less	109 (53.4)	95 (46.6)	0.178	78 (61.4)	49 (38.6)	0.51
Adequate	17 (58.6)	12 (41.4)		17 (63)	10 (37)	
Fat						
Less	93 (57.7)	68 (42.3)	0.214	82 (66.6)	41 (33.4)	0.675
Adequate	33 (45.8)	39 (54.2)		13 (41.9)	18 (58.1)	
Carbohydrate						
Less	118 (55.1)	96 (44.9)	0.123	77 (58.3)	55 (41.7)	0.25
Adequate	6 (31.5)	13 (68.5)		18 (81.8)	4 (18.2)	

## 4 Discussion

Adolescence is a transitional period from childhood to adulthood, starting from the age of 10–13 years and ending at the age of 18–22 years (15). Adolescence is a critical developmental period regarding the perception of body shape or size (19). Based on the results of the analysis, more than half of the female respondents were 14 years old in urban areas and 13 years old in rural areas.

Mid Upper Arm Circumference (MUAC) is a measurement of arm circumference midway between the tip of the elbow and the tip of the shoulder that has the potential to offer a simple alternative for assessing nutrition in adolescents (20). According to the Indonesian Ministry of Health, the size of LILA at risk of suffering from SEZ is <23.5 cm and Body Mass Index (BMI) <18.5 kg/m2 (2, 21). CED is defined as a chronic nutritional problem that results from low energy intake over many years (22). Seen in Table 1, the percentage of adolescents at risk of developing CED in rural areas is greater, in urban areas (54.0%) and rural areas (61.7%). If defined further, this figure is quite higher than the research conducted by Rahmadi, stating that as many as 44.9% of adolescents in rural areas were at risk of experiencing CED (2). Research by Wardhani, also stated that as many as 55.0% of adolescents in urban areas were at risk of being CED (23). According to Purba et al., CED can be caused by various factors such as lack of energy intake, protein intake and also family income (24) (Figure 1).

Body image refers to the way individuals see and feel about their own physical appearance. As adolescents’ bodies undergo many changes during growth, perceptions of body image can have a significant impact on their mental and emotional well-being (25). In this study, adolescents in both urban and rural areas had negative perceptions of their bodies. In Indonesia, adolescents’ body image perceptions are often influenced by the prevailing culture and social norms. Indonesian culture has diverse views on beauty and body idealization that can influence how adolescents see themselves. In addition, physiological, cognitive and sociocultural factors regarding ideal body size can also influence body image perceptions (26, 27). Adolescents may feel pressure to achieve body standards that are considered ideal by society, sometimes in unhealthy ways such as excessive dieting (28).

In the digital age, adolescents are exposed to “perfect” body images through social media platforms (29). This can create feelings of dissatisfaction with their physical appearance and increase the desire to achieve often unrealistic standards (1). In addition, family, peers and social media contribute to body shape or size dissatisfaction (30). Perceived body image and nutritional status have a strong relationship in a person’s physical and psychological health (27, 31). Body image refers to the way individuals see and feel about their physical appearance, while nutritional status reflects body health based on nutrient intake and weight. These two aspects influence each other and have a significant impact on each other. Negative body image perceptions can affect a person’s nutritional status. A person who is dissatisfied with their physical appearance may tend to take extreme measures, such as unhealthy dieting, to achieve unrealistic beauty standards. This can negatively affect their nutrient intake and nutritional status. In Hoseini’s study, it was stated that there was a significant relationship (*p* = 0.023) between perceived body image and healthy eating patterns (32). In addition, eating disorders such as anorexia nervosa and bulimia nervosa are often closely related to negative body image perceptions. Individuals with these eating disorders tend to have deep dissatisfaction with their physical appearance and attempt to control their weight in unhealthy ways, which can lead to serious nutritional problems (33).

Based on the results of the data analysis, it can be concluded that adolescents’ consumption of macronutrients is still relatively low compared to the established RDAs. In urban areas, adolescents tend to consume less macronutrients, especially energy and carbohydrate. Whereas in rural areas, adolescents tend to consume less fat intake compared to the intake of other nutrients. In line with the research of Vilhar et al., only 75% of adolescents consume carbohydrates and protein, 44% consume fat and only 10% of adolescents whose consumption is close to the recommended nutritional needs (34).

Adolescents will experience many changes, for example in physical and body changes there is an increase in muscle mass, and an increase in fat tissue in the body, as well as hormonal changes. These changes have an impact on their increasing nutritional needs. The occurrence of an imbalance between nutritional needs and intake will cause nutritional problems, both overnutrition and undernutrition problems (24). Adolescent growth and development requires complete macro and micronutrients along with increased physical activity (35). Lack of consumption of nutrient intake, especially energy and protein intake, can increase the risk of developing CED nutritional problems in adolescents (34).

## Data availability statement

The original contributions presented in the study are included in the article/supplementary material, further inquiries can be directed to the corresponding author.

## Ethics statement

The studies involving humans were approved by Mataram Health Polytechnic, Directorate General of Health Personnel, Ministry of Health of the Republic of Indonesia (LB.01.03/6/317/2023). The studies were conducted in accordance with the local legislation and institutional requirements. Written informed consent for participation in this study was provided by the participants’ legal guardians/next of kin. Written informed consent was obtained from the individual(s), and minor(s)’ legal guardian/next of kin, for the publication of any potentially identifiable images or data included in this article.

## Author contributions

CY: Conceptualization, Funding acquisition, Investigation, Methodology, Writing – original draft, Writing – review & editing. DR: Conceptualization, Data curation, Investigation, Writing – original draft. MM: Conceptualization, Data curation, Formal analysis, Software, Writing – review & editing. DS: Investigation, Writing – original draft.
